# Crystal structure of U2 snRNP SF3b components: Hsh49p in complex with Cus1p-binding domain

**DOI:** 10.1261/rna.059378.116

**Published:** 2017-06

**Authors:** Anne-Marie M. van Roon, Chris Oubridge, Eiji Obayashi, Benedetta Sposito, Andrew J. Newman, Bertrand Séraphin, Kiyoshi Nagai

**Affiliations:** 1 MRC Laboratory of Molecular Biology, Cambridge CB2 0QH, United Kingdom; 2 Equipe Labellisée La Ligue, Institut de Génétique et de Biologie Moléculaire et Cellulaire (IGBMC), Centre National de la Recherche Scientifique (CNRS) UMR 7104/Institut National de la Santé et de la Recherche Médicale (INSERM), U964/Université de Strasbourg, 67404 Illkirch, France

**Keywords:** splicing, U2 snRNP, SF3b, RNA binding, RRM

## Abstract

Spliceosomal proteins Hsh49p and Cus1p are components of SF3b, which together with SF3a, Msl1p/Lea1p, Sm proteins, and U2 snRNA, form U2 snRNP, which plays a crucial role in pre-mRNA splicing. Hsh49p, comprising two RRMs, forms a heterodimer with Cus1p. We determined the crystal structures of *Saccharomyces cerevisiae* full-length Hsh49p as well as its RRM1 in complex with a minimal binding region of Cus1p (residues 290–368). The structures show that the Cus1 fragment binds to the α-helical surface of Hsh49p RRM1, opposite the four-stranded β-sheet, leaving the canonical RNA-binding surface available to bind RNA. Hsh49p binds the 5′ end region of U2 snRNA via RRM1. Its affinity is increased in complex with Cus1(290-368)p, partly because an extended RNA-binding surface forms across the protein–protein interface. The Hsh49p RRM1–Cus1(290-368)p structure fits well into cryo-EM density of the B^act^ spliceosome, corroborating the biological relevance of our crystal structure.

## INTRODUCTION

Splicing is a process that occurs in eukaryotes to remove noncoding sequences (introns) from pre-messenger RNA (pre-mRNA) and splice together coding sequences (exons) to obtain mature RNA transcripts, which can be translated. This process is carried out by a number of protein–RNA complexes, called small nuclear ribonucleoproteins (snRNPs), and numerous non-snRNP proteins that assemble and disassemble in a stepwise manner onto the pre-mRNA to form the spliceosome (for reviews, see Wahl et al. 2009; Will and Lührmann 2011; van der Feltz et al. 2012). At the early stage of its assembly, U1 and U2 snRNPs recognize the 5′ splice site and the branch point sequence (BPS), respectively, and initiate the assembly of the spliceosome. The recent structures of an activated B-complex spliceosome (B^act^) (Rauhut et al. 2016; Yan et al. 2016), C-complex (Galej et al. 2016; Wan et al. 2016), and C*-complex (Bertram et al. 2017; Fica et al. 2017; Yan et al. 2017) have provided important insights into the mechanism of pre-mRNA splicing. The branch helix is formed when the pre-mRNA BPS pairs with U2 snRNA in U2 snRNP and is escorted into the active site of the spliceosome. In the B^act^ complex, the branch helix is bound by U2 snRNP-specific protein complexes SF3a and SF3b. Prp2p then induces the dissociation of SF3a and SF3b, allowing Prp16p and step I factors to dock the branch helix into the active site (Ohrt et al. 2012; Galej et al. 2016) for the first transesterification reaction (branching).

U2 snRNP contains U2 snRNA, seven Sm proteins, Msl1p/Lea1p (U2B′′/U2A′ in humans), and two U2 specific protein complexes SF3a and SF3b. Early negative stain EM studies of isolated human U2 snRNP revealed a bipartite domain architecture (Krämer et al. 1999). U2B′′/U2A′ bind to stem–loop IV (Price et al. 1998) and form a large 3′-domain together with seven Sm proteins bound to the Sm site. SF3b binds toward the 5′ end of U2 snRNA within 12S U2 snRNP forming the 5′-domain and allowing SF3a to bridge the two domains, forming the 17S U2 snRNP particle (Krämer et al. 1999). *Saccharomyces cerevisiae* SF3b comprises six proteins: Rse1p, Hsh155p, Cus1p, Hsh49p, Rds3p, and Ysf3p (Dziembowski et al. 2004). Human SF3b contains p14 in addition to the homologs of yeast SF3b proteins (Will et al. 2001).

CryoEM analysis of glutaraldehyde cross-linked human SF3b has been reported at better than 10 Å resolution (Golas et al. 2003). At this resolution, proteins with a known fold were fitted into the map but molecular details of their interactions were not revealed; the 22 HEAT repeats of SF3b155 were located on the outside of the complex, the RRM of p14 was fitted into density in the central cavity of the complex, and both RRMs of Hsh49p were tentatively assigned to two domains lying side-by-side on the surface of the cryoEM density (Golas et al. 2003).

High-resolution structures of the individual proteins of SF3b, and of its subcomplexes, are very valuable to gain further insights into the precise role of yeast SF3b in early stages of splicing. We reported the solution structure of Rds3p (van Roon et al. 2008), and recently the crystal structure of a core complex of human SF3b was published containing SF3b130 (Rse1p), SF3b155 (Hsh155p), SF3b14b (Rds3p), and SF3b10 (Ysf3p) (Cretu et al. 2016). In this structure the HEAT repeats of SF3b155p wrap around a bipartite scaffold comprising SF3b130, SF3b10, and SF3b14b. SF3b145 (Cus1p), SF3b49 (Hsh49p), and p14 are not present in these crystals. The solution structure of the first RRM of human SF3b49 has recently been published (Kuwasako et al. 2016).

**FIGURE 1.**
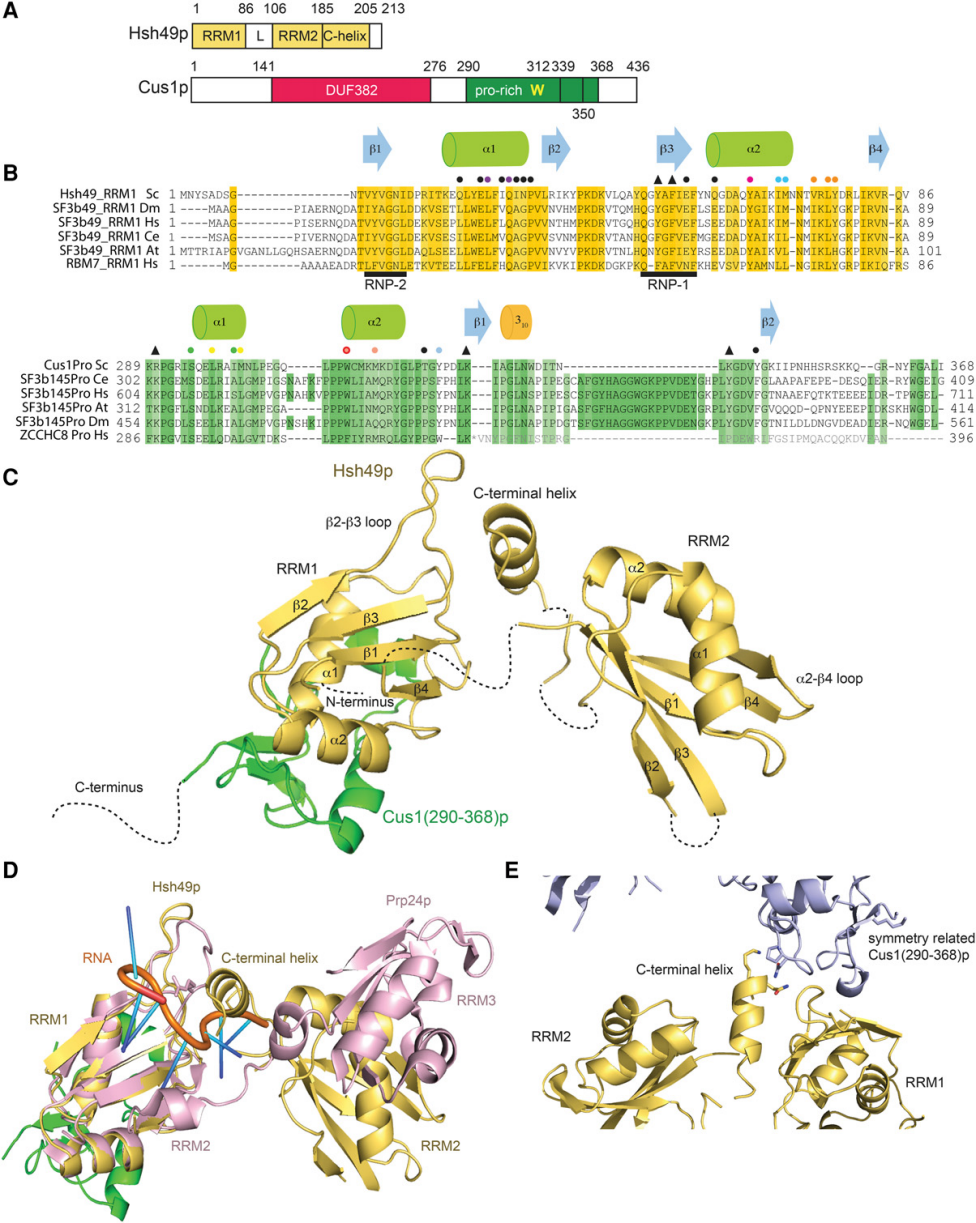
Structure of Hsh49p–Cus1(290-368)p. (*A*) Domain organization of Hsh49p and Cus1p. The proline-rich region of Cus1p (290-339) contains the absolutely conserved Trp312. (*B*) Sequence alignment of RRM1 of Hsh49p and homologs from *Drosophila melanogaster* (Dm), *Homo sapiens* (Hs), *Caenorhabditis elegans* (Ce), *Arabidopsis thaliana* (At), and Hs RBM7. Sequence alignment of the proline-rich region of Cus1p and homologs, and the proline-rich region of Hs ZCCHC8. The sequence in gray is not present in the crystal structure of RBM7–ZCCHC8^Pro^. Circles denote residues at the interface between RRM1 and Cus1(290-368)p, colored circles indicate residues that were mutated for pull-down experiments. Triangles denote residues that were mutated for RNA interaction studies. (*C*) Overall structure of the complex of Hsh49p (yellow) and Cus1(290-368)p (green) showing the arrangement of RRM1 and RRM2 with the C-terminal helix wedged in between. (*D*) Overlay of Hsh49p with RRM2 and RRM3 from Prp24p bound to U6 snRNA (4N0T). (*E*) Crystal packing between the C-terminal helix of Hsh49p and Cus1(290-368)p.

Hsh49p is an essential protein and comprises two RNA recognition motifs (RRMs); both RRMs are required for yeast viability (Igel et al. 1998). RRM1 and RRM2 of Hsh49p show high degrees of similarity (35% identical and 55% similar) to their human counterparts. However, the proline-rich C terminus, seen in metazoans, is absent in yeast Hsh49p. Cus1p, a 50 kD protein, is also an essential protein with 43% identity and 65% similarity to the human sequence in comparable regions. It contains a domain of unknown function, DUF382 (Pfam: PF04037), and a proline-rich region (Fig. 1A). Residues 121–392 of Cus1p are required for yeast viability (Pauling et al. 2000). Cus1p was originally identified as a suppressor of cold-sensitive U2 snRNA mutations, which cause defects in spliceosome assembly (Wells et al. 1996). Yeast two-hybrid experiments indicated that Hsh49p binds Cus1p via its N-terminal RRM and that the binding region within Cus1p lies between residues 229 and 311 (Pauling et al. 2000). Recently it was shown that, in early spliceosomes, Hsh49p and Cus1p crosslink to the pre-mRNA in a region upstream of the branchpoint adenosine (Schneider et al. 2015). However, in the context of isolated U2 snRNP, cross-links were observed to the 5′ end, SLI and SLIIb of U2 snRNA in humans (Dybkov et al. 2006).

In this paper, we present the crystal structures of yeast Hsh49p–Cus1(290-368)p (2.7 Å) and RRM1–Cus1(290-368)p (1.6 Å). We have also investigated the RNA-binding properties of Hsh49–Cus1(290-368)p with U2 snRNA. Finally, we fitted our complex structure into the EM density of the B^act^ spliceosome complex published recently (Yan et al. 2016). Our study has provided new insight into the structure and function of Hsh49p–Cus1p in splicing.

## RESULTS

### Mapping of the Hsh49p-binding region of Cus1p and crystallization of Hsh49p–Cus1p complexes

*S. cerevisiae* Hsh49p was readily overexpressed as soluble protein in *E. coli*, while *S. cerevisiae* Cus1p was only obtained in small quantities and is highly susceptible to degradation in vivo. When the full-length Hsh49p and Cus1p are coexpressed in *E. coli*, the Hsh49p–Cus1p dimer tends to aggregate during purification. The smallest viable fragment of Cus1p spanning residues 121–392 (Pauling et al. 2000) was stably coexpressed with Hsh49p and the complex was purified to homogeneity. However, this complex did not yield any crystals despite extensive crystallization trials so the complex was subjected to limited proteolysis using trypsin. MALDI-TOF analysis and N-terminal sequencing of the proteolyzed fragments revealed that a Cus1p fragment comprising residues 285–355 could still stably bind to Hsh49p. We were able to coexpress and purify a complex of Cus1(290–350)p with full-length Hsh49p but crystals of this complex did not diffract beyond 7 Å. We then coexpressed and purified Hsh49p with several slightly longer Cus1p fragments (286–350, 290–355, 290–360, 290–363, and 290–368). The Hsh49p–Cus1(290–368)p construct yielded small needle-like trigonal crystals, which diffracted to a maximum resolution of 2.7 Å, but at this point we were unable to solve the structure by molecular replacement or isomorphous replacement, partly due to twinning. It was previously shown that RRM1 of Hsh49p binds to Cus1p (Igel et al. 1998), so we coexpressed RRM1 of Hsh49p with the same Cus1p peptide (290–368). This complex yielded crystals that diffracted to 1.6 Å. The structure was solved by molecular replacement using the RRM of cyclophilin33 as a search model (pdb 3MDF [Hom et al. 2010]) and refined to a final model with *R*/*R*_free_ of 19.5/21.7 (see Table 1). All four molecules in the asymmetric unit are very similar with average overall RMSDs for the main chain of 0.22 Å for RRM1 and 0.42 Å for Cus1(290–368)p. The structure of the complex between full-length Hsh49p and Cus1(290–368)p could then be solved by molecular replacement using the RRM1–Cus1(290–368)p structure and RRM2 of the polyadenylate-binding protein (pdb 1CVJ [Deo et al. 1999]). Restrained twin refinement led to a final model with *R*/*R*_free_ of 18.4/23.3 (see Table 1). All three molecules in the asymmetric unit are very similar, with average overall RMSDs for the main chain of 0.45 Å for Hsh49p and 0.23 Å for Cus1(290–368)p. The higher resolution crystal structure of RRM1–Cus1(290–368)p was used for the detailed analysis of the structure of Cus1(290–368)p and the interface between RRM1 and Cus1(290–368)p. Both crystal structures overlay very well with average overall main chain RMSDs for RRM1 of 0.47 Å and 0.68 Å for Cus1(290–368)p.

**TABLE 1.**
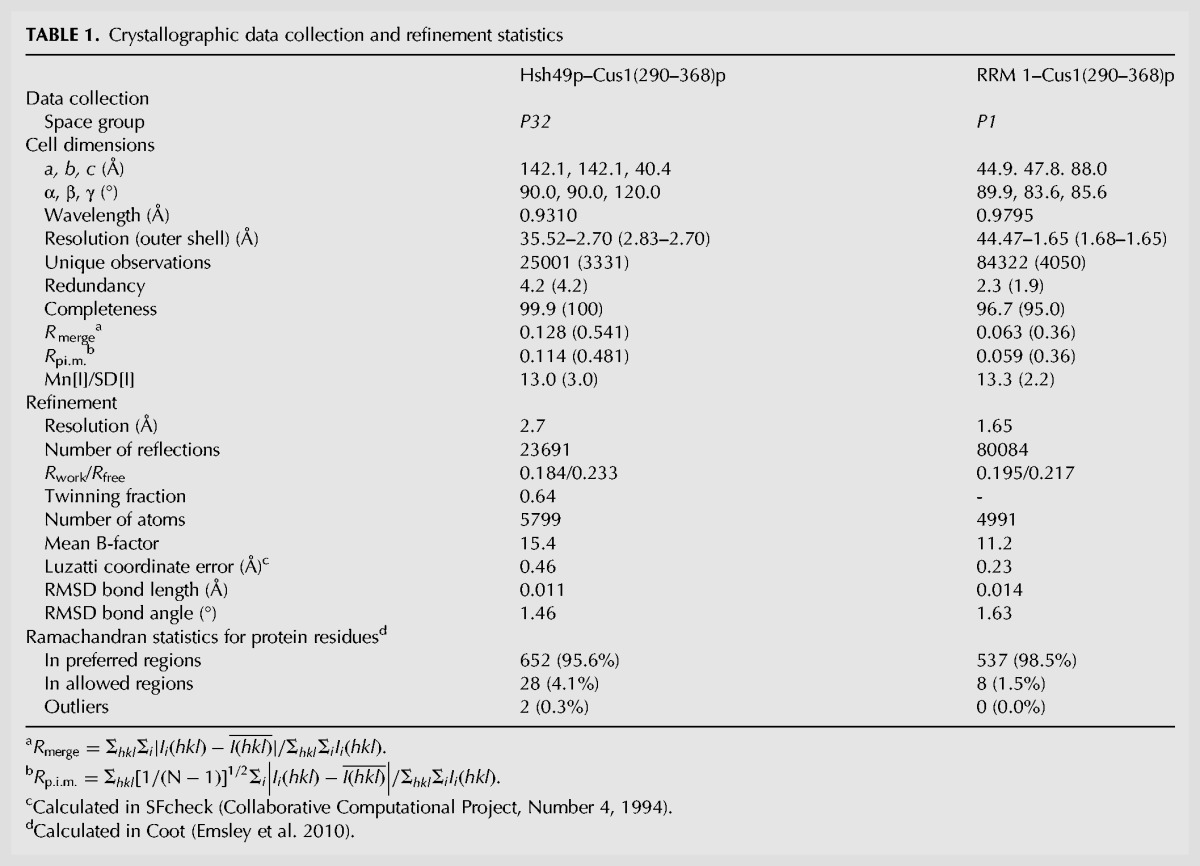
Crystallographic data collection and refinement statistics

### Structure of Hsh49p

Both RRMs of Hsh49p display the canonical RRM structure (Fig. 1C) as reviewed in Muto and Yokoyama (2012). RRM1 has three solvent exposed conserved aromatic residues, Tyr12 in the RRM's RNP2 motif, Tyr52 and Phe54 in its RNP1 motif (Figs. 1B, 6D). The first residue of RNP1 is typically basic but is a glutamine (Gln50) in RRM1 of Hsh49p and its homologs (Fig. 1B). RRM2 has two solvent-exposed, conserved aromatic residues, Phe111 in RNP2 and Tyr152 in RNP1, but is atypical in yeast as it lacks the conserved glycine at the start of β3 and the first aromatic residue of RNP1 is replaced by a cysteine (Cys150). However, there is an extra solvent-exposed tyrosine (Tyr154) within its RNP1 motif (Igel et al. 1998). Like many RRMs, RRM2 contains a β-hairpin in the loop between α2 and β4 (Cléry et al. 2008). The refined model contains residues 8–86 of RRM1, 106–185 of RRM2, and residues 187–205 of a C-terminal extension of RRM2. No density was observed for the first seven residues of RRM1, the linker connecting RRM1 and RRM2 and the last eight C-terminal residues of Hsh49p. In the absence of density for the linker it is not possible to conclude which RRM1 and RRM2 in the crystal lattice are connected covalently. The crystals do contain full-length Hsh49p as visualized by SDS–PAGE (data not shown), and the Hsh49p–Cus1(290–368)p complex behaves as a monomer in solution as seen by analytical gel filtration (data not shown). The distance between the C terminus of RRM1 and the N terminus of RRM2 of the closest pair within the asymmetric unit is 17–18 Å; to symmetry-related RRMs the distance is 30–39 Å. These distances can all be bridged by the disordered linker peptide so it is possible that a domain swap has occurred. In RRM2, the β2/β3 loop is disordered and density for one residue is missing from the linker between β4 and the C-terminal extension. This C-terminal extension of RRM2 (186–205) forms an α-helix, which folds back and wedges between RRM1 and RRM2 (Fig. 1C,D), interacting with RRM1 via the β1/α1 and α2/β4 loops and more extensively with the longer β2/β3 loop. The C-terminal helix binds to RRM2 via the β3/α2 loop.

We overlaid Hsh49p with other proteins containing tandem RRMs: hnRNP A1 (pdb 2LYV [Barraud and Allain 2013]), hnRNP L (RRM3 and 4, pdb 4QPT [Blatter et al. 2015]), CPEB1 (pdb 2MKH [Afroz et al. 2014]), Prp24p (pdb 2GO9, 2GHP [Bae et al. 2007]), and PTB domains 3 and 4 (pdb 2EVZ [Vitali et al. 2006]). The relative arrangement of the RRMs of Hsh49p in the crystal is completely different compared with these other structures. When we overlaid RRM1 of Hsh49p with RRM2 of Prp24 bound to U6 snRNA (pdb 4N0T [Montemayor et al. 2014]) on its canonical RNA binding surface, the C-terminal helix of Hsh49p would interfere with RNA binding to RRM1 in the Hsh49p–Cus1(290–368)p complex (Fig. 1D). It is possible that the arrangement of the RRMs of Hsh49p with its C-terminal helix is stabilized by crystal packing interactions. Indeed crystal contacts are observed between the bottom of the C-terminal helix and residues in the linker between α1 and α2 of a symmetry-related Cus1(290–368)p (Fig. 1E).

### Structure of Cus1(290–368)p

**FIGURE 2.**
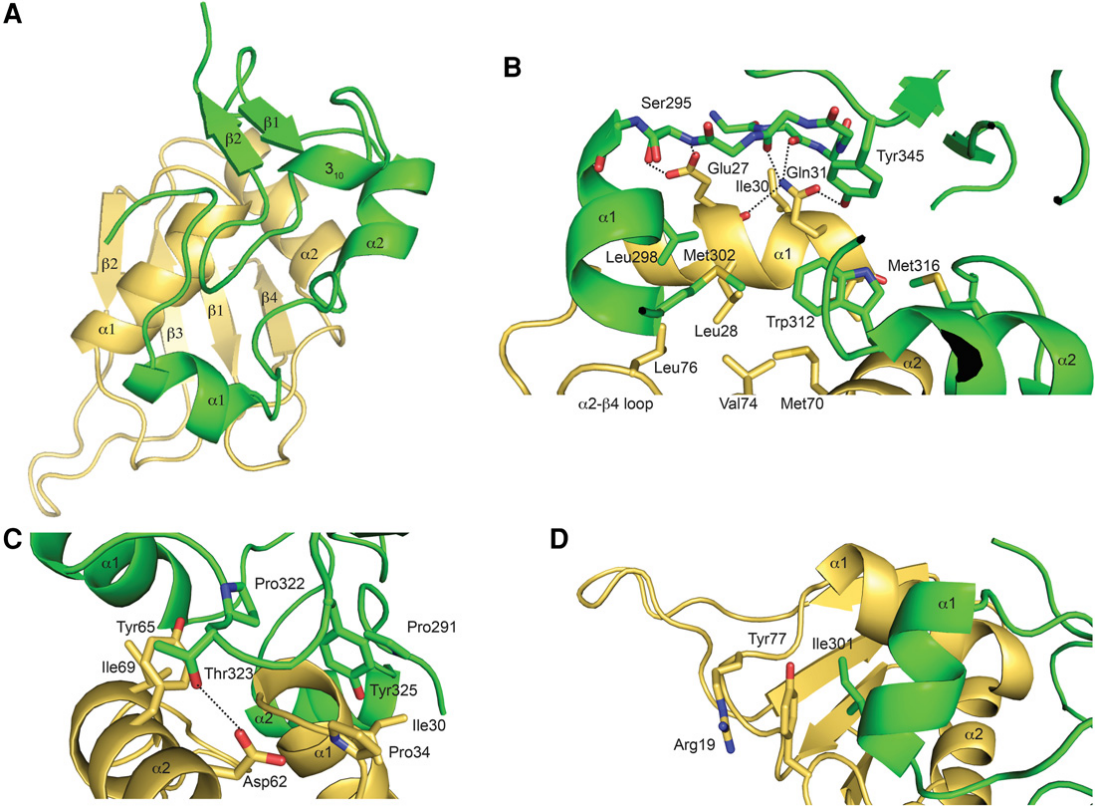
Overview of RRM1–Cus1(290–368)p interaction. (*A*) Overview of Cus1(290–368)p (green) binding to the α-helical face of RRM1 (yellow). (*B*) Close up of the *center* region of the interaction surface. Cus1(290–368)p is lying in a hydrophobic crevice on RRM1. Key interacting residues are depicted in sticks and labeled. Hydrogen bonds are depicted as dashed lines. (*C*) Close up of the face-to-face arrays of Tyr65 and Tyr325 in Hsh49p and Cus1(290–368)p, respectively. (*D*) Close up of the face-to-face array of Tyr77.

Residues 290–350 of Cus1(290–368)p are ordered and included in the model (Figs. 1 and 2). This region coincides with the smallest stable fragment that binds to Hsh49p, as shown by our limited proteolysis and coexpression experiments. The remaining 18 C-terminal residues present in our construct are disordered. The Cus1(290–368) domain is folded with most secondary structure elements located at its periphery, and it covers almost the entire α-helical side of RRM1 (Fig. 2A). The domain has two short α-helical regions, α1 (Gln296–Met302) and α2 (Trp312–Ile319), that are linked by a short anti-parallel β-sheet with a 3_10_-helix in the linker connecting the two β-strands (β1, Leu328–Ile330 and β2, Gly346–Ile348). This is further stabilized by two β-turns formed between Arg290–Arg293 and Lys341–Val344.

### Structure of the interface between RRM1 and Cus1(290–368)p

An extensive interface is created between Cus1(290–368)p and the α-helical surface of RRM1 of Hsh49p. The α1 of RRM1 is lying along a crevice on the surface of Cus1(290–368)p, making numerous hydrophobic contacts to Cus1(290–368)p via Leu28, Ile30, and Ile32. In addition, side chains of Glu27 and Gln31 form hydrogen bonds to both the main chain and side chains of Cus1(290–368)p (Fig. 2B). RRM1 α2 is located at the edge of the complex and in addition to the hydrophobic interactions via Ile69 (Fig. 2C) and Met70 (Fig. 2B) a hydrogen bond is present between the side chain of Asp62 and the side chain of Thr323 on Cus1(290–368)p (Fig. 2C). At the center of this interaction surface, a completely conserved tryptophan (Trp312), located at the bottom of α2 of Cus1(290–368)p, lies flat in a hydrophobic cleft on the surface of RRM1 created by α1, α2, and the extended linker between α2 and β4 (Fig. 2B). The hydrophobic surface of Cus1(290–368)p is extended by Leu298, Met302 (α1), and Met316 (α2). In addition, Tyr345 is lying almost perpendicular to Trp312 and points toward RRM1, making a hydrogen bond with Gln31 on α1 of Hsh49p (Fig. 2B). The interface between RRM1 and Cus1(290–368)p is stabilized by face-to-face hydrophobic stacking interactions. At the top of RRM1 α1, Tyr325 of Cus1(290–368)p is wedged in a hydrophobic pocket created by Pro291 on Cus1(290–368)p and Ile30 and Pro34 on RRM1 (Fig. 2C). On the same side of the complex, a similar interaction takes place where Tyr65 on α2 of RRM1 sits in a pocket created by Pro322 and Thr323 on Cus1(290–368)p and Ile69 on RRM1 (Fig. 2C). On the other edge of the complex, Tyr77, located in the extended linker between α2 and β4, makes stacking interactions with Ile301 on α1 of Cus1(290–368)p and Arg19 on RRM1 (Fig. 2D). All the key interacting residues on both Hsh49p and Cus1(290–368)p are highly conserved (Fig. 1B). The buried surface area of the interface is about 960 Å^2^ with a complex formation significance score of one, implying that this interface plays an essential role in complex formation (PDBe PISA [Krissinel and Henrick 2007]). This interface is highly hydrophobic in nature, which is consistent with the complex being resistant to salt concentrations of up to 1 M NaCl or 6% ammonium sulfate. In addition, there is an interaction between the β-sheet face of RRM1 and a symmetry related Cus1(290–368)p in the Hsh49p–Cus1(290–368)p crystals. The buried surface area of this interface is about 505 Å^2^, but a complex formation significance of zero implies it is likely a result of crystal packing. Indeed, this interface is not present in the RRM1–Cus1(290–368)p crystals.

### Similarities to other structures

**FIGURE 3.**
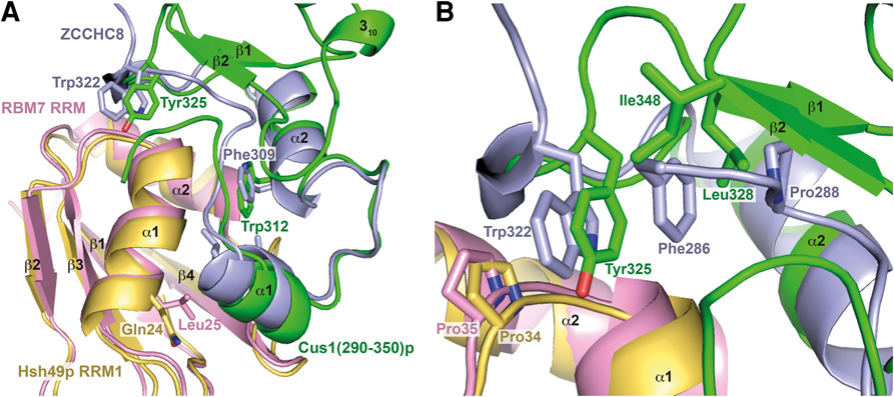
Overlay of RRM1–Cus1(290–350)p with RBM7 RRM–ZCCHC8(286–324) ([Falk et al. 2016]; pdb 5LXR). The RMSD of the main chains of the RRMs is 2.9 Å. (*A*) Overview of the interface with Hsh49p in yellow, Cus1p in green, RBM7 in pink, and ZCCHC8 in blue. Some of the variant interface residues are shown in stick representation. (*B*) Close-up of “patch 2” showing the extensive stacking array around Phe286 in the RBM7-ZCCHC8 heterodimer compared to the less striking interactions seen in this region of the Hsh49p–Cus1p complex.

Recently, the structure of the complex of exosomal accessory factors RBM7 and ZCCHC8 proline-rich region was published (Falk et al. 2016). These proteins together with hMTR4 form the nuclear exosome targeting (NEXT) complex. It was suggested that this structure would be highly homologous to the proline-rich region of SAP145 (SAP145^Pro^), the human counterpart of Cus1p. We therefore expected it to be very similar to our structure as well. Indeed, when we overlaid our structure onto the RBM7–ZCCHC8 complex, the RMSD of the main chain was 2.9 Å for the RRMs and 5.6 Å for Cus1(290–368)p between residues 290 and 324 (Fig. 3A). Interestingly, residues forming the interaction hotspot involving Hsh49p α1, the extended linker between α2 and β4 and α1 of Cus1(290–368)p (denoted patch 1 in the RBM7–ZCCHC8 structure) are highly conserved except for Leu25, which is replaced by Gln24 in Hsh49p (Fig. 3A). Its aliphatic side chain still provides hydrophobicity, but in addition a hydrogen bond with Gln297 of Cus1p further stabilizes the interaction. Another interesting difference is the replacement of the completely conserved Trp312 in Cus1p for Phe309 in ZCCHC8 (Fig. 3A). Most residues involving Hsh49p α2 and the loop following α1, the N terminus of Cus1(290–368)p as well as α2 and the 3_10_ helix (denoted patch 2 in the RBM7–ZCCHC8 structure), are highly conserved with one remarkable exception: Phe286 in ZCCHC8 is sandwiched by Pro288, Trp322, and Pro35 of RBM7 in a face-to-face stacking array (Fig. 3B). In our structure, the N terminus of Cus1(290–368)p is pushed away by β2 in Cus1(290–368)p, and the position of Phe286 is taken by Leu328 and Ile348. Interestingly, Phe286 is conserved in ZCCHC8 homologs but not in Cus1p from different species, where it is replaced by a basic residue (Fig. 1B). Trp322 is replaced by Tyr325 in Cus1(290–368)p, which only stacks face to face with Pro34 of Hsh49p and in addition forms a hydrogen bond with the main chain carbonyl of Ile30 creating extra stability.

In addition, solution structures of both RRMs of the human homolog of Hsh49p, SF3b49, have been deposited to the protein databank (pdb codes 1X5U and 1X5T). The ordered regions of *S. cerevisiae* RRM1 and RRM2 align very well with their human homologs, with overall RMSDs for the main chain of 1.6 Å and 2.4 Å, respectively. In a recent paper, the mode of interaction between SF3b49 RRM1 and a fragment of the proline-rich region of SF3b145, residues 598–631 (corresponding to Cus1p residues 279–311) was studied by NMR (unreleased pdb) (Kuwasako et al. 2016). A model was proposed based on chemical shift mapping and NOESY measurements in which SF3b145 residues 607–616 form an α-helix upon interaction with RRM1, and this helix interacts with RRM1 of SF3b49 via α1 and Tyr80 (equivalent to Tyr77 in our structure). This is consistent with our structure, though the α-helix in our structure does not start until Gln296 (equivalent residue 611 in SF3b145). As the sequences are highly homologous (46% identity for the proline-rich domain), it is very likely that the structures of the whole domain will be highly similar.

### Pull-down experiments

**FIGURE 4.**
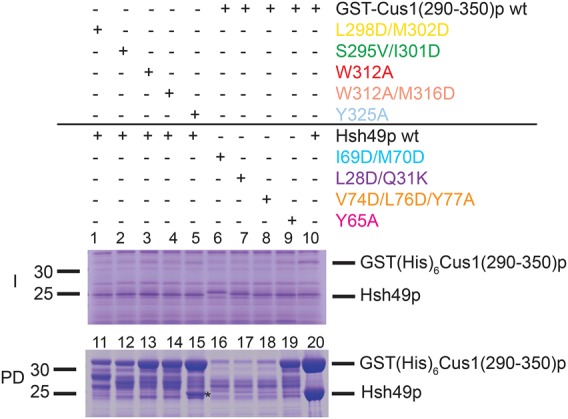
Coexpression of Hsh49p and GST(His)_6_Cus1(290–350)p wild-type or mutant proteins and pull-down experiment. The proteins were coexpressed in *E. coli* and whole-cell contents are shown in the Input gel (I). Similar levels of Hsh49 are seen in all lanes but levels of tagged Cus1(290–350)p vary. Cells were lysed and after centrifugation the supernatant was mixed with Ni-NTA resin. Protein that remained bound to the resin after washing is shown in the Pulldown gel (PD). Only for complexes containing both wild-type components were stoichiometric levels of Hsh49p recovered from the pulldown; otherwise only in lane *15* [Cus1(290–350)p Y325A mutant] are significant levels observed. We attribute the lack of full-length tagged Cus1(290–350)p in many Input and Pulldown lanes to degradation of the Cus1(290–350)p, which is protease-sensitive when not bound to Hsh49p.

We have designed mutations and performed pull-down experiments in vitro to test which residues are important for the interaction between Hsh49p RRM1 and Cus1(290–350)p. Glutathione–hexahistidine-tagged Cus1(290–350)p constructs were coexpressed with untagged Hsh49p and then bound to nickel beads, washed, and eluted. The results of the pull-down experiments are shown in Figure 4. Interestingly, only the Tyr325Ala mutant of Cus1(290–350)p was able to capture significant amounts of Hsh49p. Tyr325 is sandwiched with Pro291 on Cus1p and Pro34 on Hsh49p on the periphery of the complex (Fig. 2C). All other Hsh49p or Cus1(290–350)p mutants, which are located near the center of the interaction surface, disrupted the complex. As shown on the gel, when Cus1(290–350)p does not bind Hsh49p it is unstable and gets degraded. This is already clear from the input gel (Fig. 4, lanes 1–10); expression levels are similar, as can be seen from constant levels of Hsh49p, whereas the amount of Cus1(290–350)p varies. It is most noticeable in lanes 6–8 and 16–18 where only small amounts of intact GST–Cus1(290–350)p are present.

### RNA-binding studies

**FIGURE 5.**
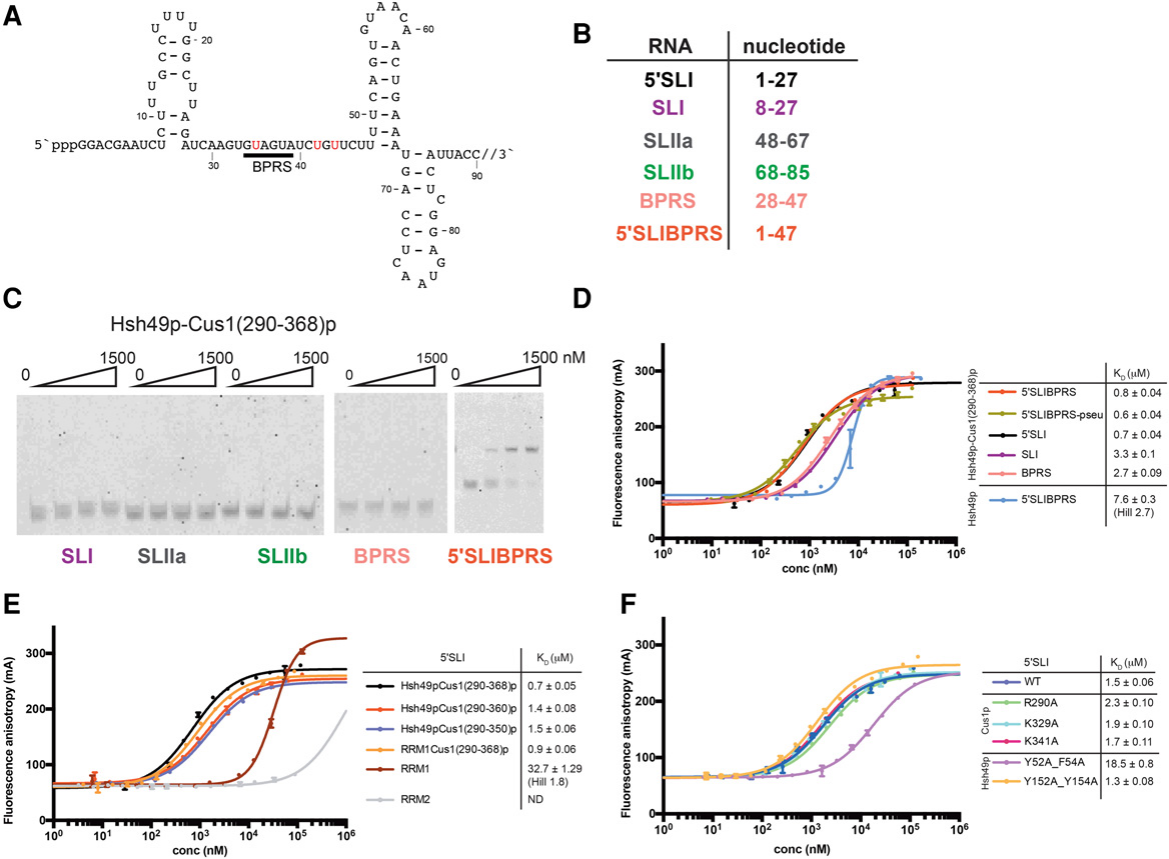
RNA-binding studies of Hsh49p–Cus1(290–368)p with different 5′ end U2 snRNA oligos. (*A*) 5′ end of *S. cerevisiae* U2 snRNA. The pppG at the 5′ terminus is a result of the in vitro transcription, and the nucleotides denoted in red are post-transcriptionally modified to pseudouridine in vivo. (*B*) Summary of 5′ U2 snRNA oligos used for bandshift assays. (*C*) Bandshift of different 5′ U2 snRNA oligos. (*D*) Fluorescence anisotropy measurements of different RNA oligos with Hsh49p–Cus1(290–368)p or Hsh49p. In the absence of Cus1(290–368)p, Hsh49p binds significantly weaker. The error bars represent the SD of each data point calculated from three independent fluorescence anisotropy measurements. (*E*) Fluorescence anisotropy measurements of different Hsh49p–Cus1p constructs with 5′SLI oligo. As in *D*, it is immediately obvious that the presence of Cus1p proline-rich domain enhances the affinity. The data of RRM1 had to be fitted with a Hill coefficient of 1.8; in addition the higher maximum anisotropy shows that multiple RRMs could be binding to the RNA. (*F*) Fluorescence anisotropy measurements of mutants of Hsh49p or Cus1(290–350)p with 5′SLI. Mutation of the canonical RNA-binding residues of RRM1 significantly impair RNA binding. In addition, the Cus1p Arg290Ala mutant also decreases the RNA-binding affinity.

**FIGURE 6.**
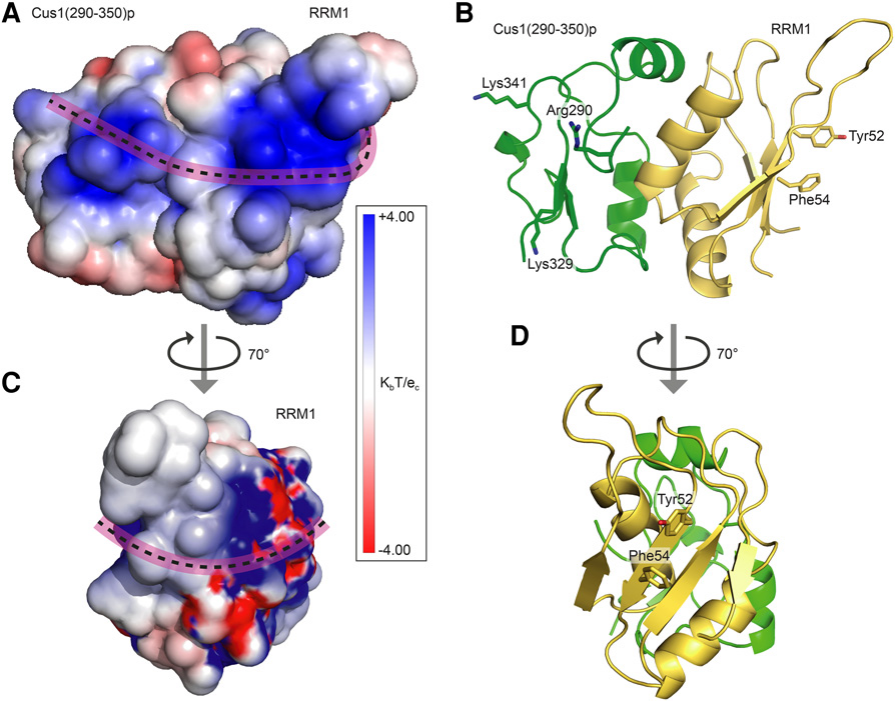
Electrostatic surface of Hsh49p RRM1–Cus1(290–350)p. Crystals contained Cus1(290–368)p but residues 351–368 were disordered. (*A*) The electrostatic surface of the interface displays an extended positively charged region (blue). The surface potential was calculated using the program PDB2PQ (Dolinsky et al. 2007) and APBS (Baker et al. 2001). A pink dotted line represents where RNA may bind this basic region as well as the β-sheet of RRM1 on the *right-hand* edge, which is the canonical RNA-binding surface, to partially explain Cus1p's contribution to Hsh49p RNA binding. (*B*) Cartoon representation of the surface shown in *A*, upon which the residues mutated in the RNA-binding studies are indicated. (*C*) Rotated view of *A* showing the canonical RNA-binding surface. A possible RNA-binding site is indicated by the pink dotted line. (*D*) Cartoon representation of the surface shown in *C*.

Yeast Hsh49p has been shown to shift a U2 snRNA construct containing the regions required for viability in *S. cerevisiae* (Igel et al. 1998). Human SF3b49p has been reported to crosslink the 5′ region of U2 snRNA in 17S U2 snRNP (Dybkov et al. 2006). In order to see if Hsh49p can bind the 5′ end of yeast U2 snRNA we designed a number of short RNA oligos representing 5′ end elements of U2 snRNA for a bandshift assay: stem–loop I (SLI), 5′ stem–loop I (5′SLI), stem–loop IIa (SLIIa), stem–loop IIb (SLIIb), branchpoint recognition site (BPRS), and BPRS including 5′ end and SLI (5′SLIBPRS) (Fig. 5A,B). Interestingly, Hsh49p alone did not shift any of the RNA oligos (data not shown), whereas in a complex with Cus1(290–368)p it did bind to 5′SLIBPRS (Fig. 5C). However, the complex did not shift any of the other RNA oligos in the bandshift assay (Fig. 5C). To measure binding affinities of Hsh49p and Hsh49p–Cus1p complexes for the different RNA oligos, we performed fluorescence anisotropy studies with 3′ end labeled RNA. Most data could be fitted to a single binding site model. However, we had to include a Hill coefficient to fit the Hsh49p and RRM1 data. We found that Hsh49p–Cus1(290–368)p complex binds an order of magnitude more strongly to 5′SLIBPRS than Hsh49p alone (compare the red curve with the light blue curve in Fig. 5D). In addition, RNA oligos containing the 5′ end of U2 snRNA bind significantly more strongly to Hsh49p–Cus1(290–368)p complex. The presence of the conserved pseudouridines around the BPRS (Massenet et al. 1999) did not have a large effect on binding (5′SL1BPRS-pseu, green curve, Fig. 5D). Next we tested different protein constructs with 5′SLI to establish which domains are important for the interaction. We found that RRM1 is responsible for Hsh49p's RNA interaction (Fig. 5E), albeit with low affinity (33 µM). Binding of RRM2 was unquantifiable under the conditions used. Similar to full-length Hsh49p, when RRM1 is in complex with Cus1(290–368)p, the affinity increased dramatically (0.9 µM), suggesting the proline-rich domain of Cus1p also interacts with RNA. Indeed, when we deleted the last eight residues in Cus1(290–368)p, including Tyr363 and Phe364, which is replaced by Trp in other homologs (Fig. 1B), the affinity dropped twofold (Fig. 5E). Deletion of the next 10 residues did not have any additional effect. Unfortunately we cannot see these C-terminal 18 residues in our structure as they are disordered, but when we examined the electrostatic surface of Hsh49p–Cus1(290–350)p, we found a positively charged region extending beyond the canonical RNA-binding site along the side of the complex (Fig. 6). This could in addition explain why Hsh49p binds RNA around one order of magnitude tighter when it is in complex with Cus1(290–368)p. In order to test this hypothesis we designed and coexpressed Hsh49p mutants Tyr52Ala–Phe54Ala (RRM1 RNA-binding mutant) and Tyr152Ala–Tyr154Ala (RRM2 RNA-binding mutant) with wild-type Cus1(290–350)p as well as Cus1(290–350)p basic patch mutants Arg290Ala, Lys329Ala, and Lys341Ala in complex with wild-type full-length Hsh49p. From the graph in Figure 5F it is immediately clear that the RRM1 mutant has drastically decreased RNA affinity, about 10-fold compared to the wild type. As expected, the RRM2 mutant showed no effect, whereas there was a slight but significant decrease in the Cus1(290–350)p-Arg290Ala mutant, further supporting our hypothesis that Cus1(290–368)p directly contacts the RNA as well. Figure 6A and C show a possible path of the RNA, extending from the canonical RNA binding site of RRM1 to the basic patch of Cus1(290–350)p.

### Fitting of Hsh49p–Cus1(290–368)p complex into B^act^ EM density

**FIGURE 7.**
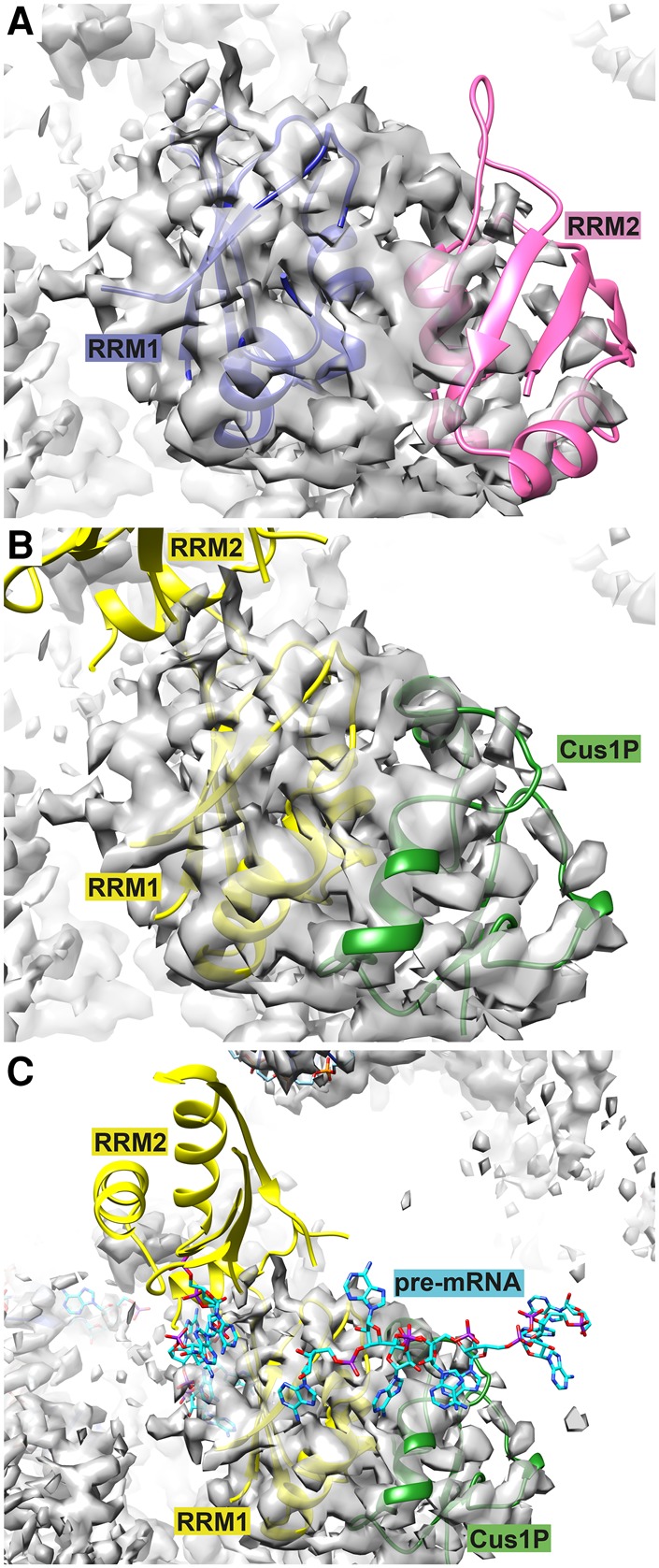
Comparison of Hsh49 modeled into EM density of yeast activated spliceosome (B^act^) ([Yan et al. 2016], EMD-9524) with the crystal structure presented here. (*A*) Originally, RRM1 (blue) and RRM2 (pink) of Hsh49 were modeled side-by-side into the map ([Yan et al. 2016], pdb 5GM6). (*B*) The Hsh49p–Cus1(290–368)p structure was fitted into the EM density by superposition on RRM1 of the model of Yan and colleagues (RMSD 1.23 Å). Cus1(290–368)p (green) fits well into the density ascribed to RRM2, but the RRM2 from the crystal structure (yellow) would locate in an empty region of the map. (*C*) RRM2 may fit in unassigned density seen in the *bottom left* of this panel. Some of the pre-mRNA has been modeled (Yan et al. 2016) and the nucleotides seen to the *left* of RRM1 partially occupy unassigned density across the face of its β-sheet. There is no clear density for the nucleotides that lie across the edge of RRM1 and Cus1(290–368)p.

Recently, the coordinates and EM maps of an activated B-complex spliceosome (B^act^) were released (pdb 5GM6, EMD-9524) (Yan et al. 2016), and we have overlaid our crystal structure with the B^act^ model and fitted it into the electron density map. Interestingly, RRM1 fits very well into the density that was assigned as Hsh49p RRM1, but this fit would then position the Cus1(290–368)p fragment into the density that was assigned as RRM2 in the B^act^ complex. The Cus1(290–368)p model fits this density much better than RRM2 (Fig. 7B), and in addition its N terminus now perfectly links to the C terminus of Cus1(131–289)p modeled in the EM density. This shows that the interface between Hsh49p and Cus1(290–368)p we found is indeed biologically relevant. However, this implies that RRM2 has to be placed elsewhere in the structure. There is unassigned density present to the left of RRM1 (Fig. 7C), which is close enough to be RRM2. Consequently, RRM2 is now located near stem–loop IIb of U2 snRNA, and indeed a crosslink has been observed between SAP49p and stem–loop IIb in human 17S U2 snRNP and purified A and B complexes (Dybkov et al. 2006). However, we did not detect any binding between RRM2 and SLIIa or SLIIb by fluorescence anisotropy (data not shown). The flexible linker between RRM1 and RRM2 is about 20 amino acids long and should allow independent movement of the two RRMs, indicating that the relative orientation of both RRMs held together by the C-terminal helix in the crystal structure was indeed most likely due to crystal packing interactions.

## DISCUSSION

In this article, we report the structure of Hsh49p in complex with the Hsh49p-binding domain of Cus1p. The entire α-helical face of RRM1 is cradled by Cus1(290–368)p, leaving the opposite β-sheet side available to bind another protein partner or RNA. RRM2 is not involved in Cus1p binding, which is in agreement with previous pull-down studies (Igel et al. 1998). The ordered part of Cus1(290–368)p in the crystal, residues 290–350, corresponds to the minimum binding domain of Cus1p able to bind Hsh49p, as determined by limited proteolysis and coexpression studies.

**FIGURE 8.**
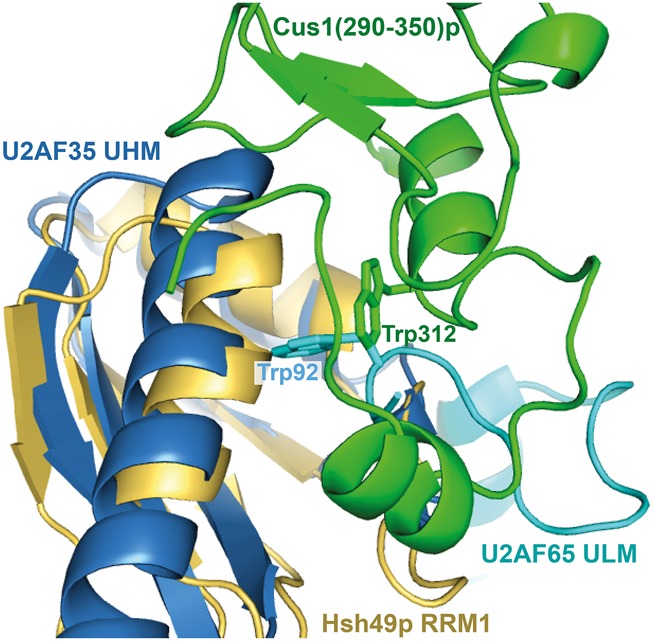
Comparison of Hsh49p RRM1–Cus1(290–350)p interface with the interaction seen in the UHM–ULM complex of U2AF35–U2AF65 (pdb 1JMT). The structures are superposed on the RRMs with an RMSD of 1.91 Å. In both cases a tryptophan is interacting with the α-helical side of the RRM but the Cus1 Trp312 side chain is not inserted in a cleft between the α-helices like the ULM Trp92 of U2AF65. Apart from the general proximity of the tryptophans, there is no resemblance between Cus1(290–350)p and U2AF65 ULM.

A completely conserved tryptophan (Trp312) is located at the center of the interaction surface between Hsh49p and Cus1(290–368)p. We have shown in our pull-down studies that Trp312 is required to bind Hsh49p. A critical role has been found for tryptophan residues in the interactions between RRMs that contain a U2AF homology motif (UHM) and their ligands, which contain a UHM ligand motif (ULM). The ULM tryptophan is inserted into a hydrophobic pocket between the two α-helices of the UHM-containing RRM (Fig. 8), as reviewed in Kielkopf et al. (2004). This pocket is surrounded by negatively (or occasionally positively) charged residues (Elantak et al. 2010) with oppositely charged residues on the ligand surrounding the tryptophan. In addition, an Arg-X-Phe motif can be found at the bottom of α2 and the loop connecting α2–β4 on the UHM. When we overlaid our structure with the U2AF35–U2AF65 complex (pdb 1JMT [Kielkopf et al. 2001]), a canonical UHM–ULM complex, the conserved tryptophan Trp312 side chain in our structure is rotated by about 90°. It is not buried in a hydrophobic pocket as seen in the UHM–ULM interface but lies flat in a hydrophobic crevice on the surface of RRM1 (Fig. 8). Apart from the conserved tryptophan, our structure lacks the other important UHM–ULM features. Instead, it represents a recently discovered novel type of RRM protein interaction (Falk et al. 2016). In our structure an extensive and conserved hydrophobic interface is present. Mutations of residues at the center of the interface completely disrupted the complex as we found in our in vitro pull-down assays (Fig. 4). Our interface is similar to that recently observed in the RBM7-ZCCHC8 structure (Fig. 3; Falk et al. 2016). It was suggested that the mutually exclusive interaction of RBM7 with ZCCHC8 and SAP145^Pro^ targets intronic sequences to the exosome. No homologs of the NEXT complex proteins were found in budding yeast, so it is unlikely that Cus1p is involved in a similar mechanism.

We found that the RNA-binding properties of Hsh49p originate from RRM1. Mutation of the canonical RNA-binding residues of RRM1 (Tyr52 and Phe54) severely diminished binding (Fig. 5F). Cus1p proline-rich domain enhances the binding to RNA and so does the presence of the 5′ end of U2 snRNA. Neither the disordered linker between RRM1 and RRM2 nor the C-terminal α-helix contribute to U2 snRNA oligo binding as Hsh49p–Cus1(290–368)p and RRM1–Cus1(290–368)p behave similarly in the fluorescence anisotropy experiments (Fig. 5E). An extended positive surface is present beyond the canonical RNA binding site of RRM1 when it is in complex with Cus1(290–350)p (Fig. 6A). Based on our fluorescence anisotropy data from different Hsh49p–Cus1p constructs and mutants, we suggest that enhancement of the RNA interaction by the Cus1(290–368)p domain is due to its direct interaction with the RNA. Indeed, deletion of the last 18 residues of Cus1(290–368)p lowered the affinity twofold (Fig. 5E), and mutation of conserved basic residue Arg290 to Ala of Cus1p(290–350)p had an additional small, but still significant effect (Fig. 5F). We suggest a possible path of the RNA along the surface of RRM1 and Cus1p (Fig. 6A,C). Enhancement of RNA interaction of an RRM bound to a protein on its α-helical surface has also been observed in the case of U2B′′–U2A′, where the presence of a U2A′ leucine rich repeat with a basic patch was required for U2B′′ RRM to bind its cognate RNA (Price et al. 1998).

We were able to fit the RRM1–Cus1(290–368)p part of the Hsh49p–Cus1(290–368)p structure into the cryoEM map of the B^act^ complex (Yan et al. 2016), showing that the structure of our complex is of biological significance and that the relative arrangement of the RRMs is due to crystal packing (Fig. 7). Unfortunately, the EM structure did not provide better insight into the RNA-binding region of Hsh49p–Cus1(290–368)p. Pre-mRNA was modeled along the top of RRM2 [which we have now reassigned as Cus1(290–368)p]; however, there is no density present for this RNA (Fig. 7C), so no conclusions can be drawn. In addition, there appears to be some extra density across the RNA-binding surface of RRM1; pre-mRNA, upstream of the branchpoint adenosine, has been modeled into it (Yan et al. 2016). Although it is only roughly fitted, this would be in agreement with the recent crosslinking study (Schneider et al. 2015). It is very likely that U2 snRNP proteins will interact differently with U2 snRNA in the 17S U2 snRNP and the spliceosome. The 5′ end of U2 snRNA forms a duplex with U6 snRNA (stem–loop II) upon integration into the spliceosome. Hsh49p–Cus1p might play a role in protecting the 5′ end of U2 snRNA until it is ready to make this interaction.

CryoEM has provided unprecedented insight into the molecular mechanism of pre-mRNA splicing (for review, see SH Scheres and K Nagai, unpubl.). Crystal structures of its components were either essential or greatly facilitated initial interpretation of the cryoEM density maps of the spliceosome. As exemplified here, crystal structures and functional studies of the components still prove invaluable in building complete atomic models of large complexes and understanding the interactions between the components that make up the entire assemblies.

## MATERIALS AND METHODS

### Cloning and overexpression of Hsh49p and Hsh49p–Cus1p complexes

Coding sequences for yeast *Hsh49* and *Cus1* were amplified by PCR from yeast genomic DNA. The *Hsh49*, *RRM1*, or *RRM2* sequences were cloned between the BamHI and EcoRI sites of the pRK172 vector (McLeod et al. 1987), which contained either a hexa-histidine and tobacco etch virus (TEV) protease site or a glutathione hexa-histidine and TEV site in frame and just upstream of the BamHI site. An NheI site was created just upstream of EcoRI, allowing the transfer of *Cus1* gene fragments, including a Shine–Dalgarno sequence from a pUC2 vector cut with XbaI and EcoRI. For the pull-down studies, a *Cus1(290–350)* gene fragment was cloned in frame with a glutathione (GST) hexa-histidine and TEV site and untagged *Hsh49*. Hsh49p and Hsh49p–Cus1(290–368)p complexes were expressed in BL21(DE3)RIL CodonPlus cells (Stratagene). The His-tagged and GST-His-tagged protein or complexes were expressed for 4 h at 37°C after induction with 0.5 mM IPTG. Harvested cells were resuspended in Nickel-NTA buffer A (20 mM Tris-HCl, pH 7.4, 500 mM Urea, 500 mM NaCl, 25 mM imidazole, and 5 mM β-mercaptoethanol), containing cOmplete protease inhibitor cocktail (Roche) and lysed by sonication. The lysate was cleared by centrifugation, the supernatant loaded on Nickel-NTA agarose (Qiagen) equilibrated with Nickel-NTA buffer A, and the protein was eluted by a 25–500 mM linear gradient of imidazole (Ni-NTA buffer B). Peak fractions were incubated overnight with His-tagged TEV protease at room temperature while dialyzing against Ni-NTA buffer A. After complete cleavage the sample was reloaded on Ni-NTA agarose to remove His-tagged TEV protease, cleaved His-tag, and minor protein contaminants. The flowthrough containing Hsh49p or Hsh49p–Cus1(290–368)p was dialyzed into heparin buffer A (20 mM Tris-HCl, pH 7.4, 200 mM NaCl, and 5 mM β-mercaptoethanol), loaded onto heparin sepharose (GE Healthcare), and eluted with a 0.2–1 M linear gradient of NaCl. Peak fractions were pooled, and Hsh49p or Hsh49p–Cus1(290–368)p was concentrated and buffer exchanged into heparin buffer A using an Amicon-Ultra-15 concentrator (Millipore) with an exclusion size of 10 kDa.

### Limited proteolysis

HisHsh49p–Cus1(114–392)p (40 μM) was incubated with trypsin (10 µg/mL final concentration) for 10 min at 22°C, PMSF (1 mM final concentration) was added to block the reaction, and 50 µL Ni-NTA resin was added to pull-down His-tagged Hsh49p with the digested Cus1p fragments. After elution with Ni-NTA buffer B, the protein mixture was run on a 4%–20% SDS-PAGE gradient gel and blotted for N-terminal sequencing, or the Coomassie-stained bands were cut out for MALDI-TOF analysis.

### Crystallization and structure determination

Crystals of Hsh49p–Cus1(290–368)p and RRM1–Cus1(290–368)p were grown using the sitting drop vapor diffusion method at 20°C. Protein solution at a concentration of 10–15 mg/mL was mixed with one volume of reservoir solution containing 2.45–2.65 M NaCl in 0.1 M sodium acetate pH 4.8–4.9 for Hsh49p–Cus1(290–368)p or 18% PEG4K, 0.1 M Tris-HCl pH 8.5, 45 mM Li_2_SO_4_ for RRM1–Cus1(290–368)p. Crystals suitable for diffraction studies grew within 10 d after streak seeding for Hsh49p–Cus1(290–368)p and 3 d for RRM1–Cus1(290–368)p. A single crystal was transferred to a cryoprotectant solution, which contained the respective reservoir solution and 15%–20% glycerol, prior to flash-cooling by plunging into liquid nitrogen. Crystallographic data were collected at the European Synchrotron Radiation Facility beamline ID14-3 for Hsh49p–Cus1(290–368)p and beamline IO2 at Diamond Light Source for RRM1–Cus1(290–368)p. Data were indexed, scaled, and merged using the automated data reduction system xia2 using CCP4, distl, labelit, pointless, scala, and xds (Collaborative Computational Project, Number 4 1994; Evans 2006; Zhang et al. 2006; Kabsch 2010; Winter 2010).

The structure of RRM1 within the RRM1–Cus1(290–368)p complex was determined by molecular replacement using the program BALBES (Long et al. 2008) with the RRM of cyclophilin33 as a search model (pdb 3MDF). This solution was verified with SHELXE (Thorn and Sheldrick 2013), followed by automated building of Cus1(290–368)p by ARP/wARP into the electron density map (Langer et al. 2008); model building was carried out in Coot (Emsley et al. 2010). The structure was refined using Refmac5 (Murshudov et al. 2011) and the maps were improved using ARP/wARP (Perrakis et al. 1997). The structure of Hsh49p–Cus1(290–368)p was solved by molecular replacement using RRM1–Cus1(290–368)p as a search model with PHASER (McCoy et al. 2007), searching for three molecules in the asymmetric unit. After a partial solution was obtained, the three RRM2 portions could be located one by one using RRM2 of the polyadenylate binding protein (pdb 1CVJ [Deo et al. 1999]) as a search model. In between each molecular replacement step the model was refined with Refmac5 using jelly body with twin refinement. The model was optimized using restrained and twin refinement with Refmac5, map improvement with ARP/wARP, and manual rebuilding. Both structures were submitted to the PDB-redo server (Joosten et al. 2011) to finalize the refinement.

### Pull-down experiments

Primers to introduce mutations in the *Cus1(290–350)* and *Hsh49* gene fragments were designed and used as described in García-Nafría et al. (2016). Glutathione hexa-histidine Cus1(290–350)p constructs were coexpressed with Hsh49p as described above except that cells were grown at 15°C after induction. After harvesting, cells were frozen, thawed, and resuspended in Pulldown buffer (20 mM Tris-HCl, pH 7.4, 0.5 M NaCl, 5 mM 2-mercaptoethanol with one cOmplete EDTA-free protease inhibitor cocktail tablet [Roche] per 100 mL) then lysed by sonication. After centrifugation at 72,000*g* for 30 min at 4°C, 800 µL of supernatant was mixed with 10 µL 2 M imidazole-HCl, pH 7.4 and 30 µL Ni-NTA resin. After 2 h incubation with gentle mixing at 4°C, the resin was pelleted by centrifugation and washed twice with 300 µL Wash buffer (Pulldown buffer with 25 mM imidazole-HCl), then resuspended in 100 µL SDS-PAGE loading buffer, heated for 2 min at 90°C, and the protein released was analyzed by SDS-PAGE.

### In vitro RNA transcription and purification

Synthetic DNA templates for RNA transcription were ordered from Sigma-Aldrich and RNA was transcribed by standard methods (Milligan et al. 1987). RNA products were purified on a Resource Q column (GE Healthcare) equilibrated in 20 mM Tris-HCl pH 8.0, 50 mM NaCl, 8 mM MgCl_2_, 1 mM DTT and eluted with a linear gradient of 50–700 mM NaCl. Peak fractions were visualized on a 15% polyacrylamide denaturing gel containing 8 M Urea in TBE (89 mM Tris-Borate, 2 mM EDTA, pH 8.3), pooled and exchanged and concentrated in water. Alternatively, the RNA products were purified on a 20% polyacrylamide denaturing gel containing 8 M Urea in TBE, visualized by UV shadowing, excised from the gel, passively eluted, and exchanged and concentrated in water.

### RNA protein-binding assays

RNAs used in the binding studies were 3′ end labeled with fluorescein, as described in Hardin et al. (2015). Electrophoretic mobility shift assay (EMSA) was used to qualitatively study the interaction of U2 snRNA-related RNAs with Hsh49p and Hsh49p–Cus1(290–368)p. Labeled RNA (10 nM) was mixed with increasing amounts of protein (0–1.5 µM) in RNA-binding buffer (20 mM K-Hepes, pH 7.5, 125 mM KCl, 1 mM EDTA, 5 mM DTT, 0.01% Igepal) in 10 µL final reaction volume in the presence of *E. coli* tRNA (500 ng) as the competitor. Binding reactions were incubated at 22°C for 20 min. Protein–RNA complexes were resolved on 12% polyacrylamide native gels in 0.5× TBE at 5 W for 75–90 min at 4°C. The gels were visualized on a Typhoon imager.

### Fluorescence anisotropy

Quantitative binding studies were carried out by fluorescence anisotropy. Labeled RNA (5 nM) was mixed with increasing amounts of protein (0–100 µM) and *E. coli* tRNA (100 ng) in RNA-binding buffer in 20 µL final reaction volume. The binding reactions were incubated at 22°C for 20 min, and the change in fluorescence anisotropy with increasing protein concentration was measured on a Pherastar with filter settings FP 485 520 520 for fluorescein. The data were analyzed by nonlinear regression curve fitting using Prism 6 software. Measurements were carried out in triplicate.

## DATA DEPOSITION

Atomic coordinates and structure factors have been deposited in the Protein Data Bank, www.wwpdb.org [pdb codes 5LSB for Hsh49p–Cus1(290–368)p and 5LSL for RRM1–Cus1(290–368)p].
